# Prevalence of asthma, allergic rhinitis and eczema in elementary schools in Sari (Iran)

**Published:** 2012

**Authors:** Javad Ghaffari, Iraj Mohammadzadeh, Alireza Khalilian, Houshang Rafatpanah, Hamid Mohammadjafari, Ali Davoudi

**Affiliations:** 1 Department of Pediatrics, Mazandaran University of Medical Sciences, Sari, Iran.; 2 Non-Communicable Pediatrics Diseases Research Center, Amirkola Children’s Hospital, Babol University of Medical Sciences, Babol, Iran.; 3 Department of Statistics, Mazandaran University of Medical Sciences, Sari, Iran.; 4 Department of Immunology, Mashhad University of Medical Sciences, Mashhad, Iran.; 5 Mazandaran University of Medical Sciences, Sari, Iran.

**Keywords:** Elementary school, Asthma, Allergic rhinitis, Eczema.

## Abstract

**Background:**

Allergic diseases including asthma, allergic rhinitis (AR) and eczema are common chronic diseases in children. The purpose of this study was to determine the prevalence of asthma, AR and eczema in Sari, Iran.

**Methods:**

This study was carried out on all elementary schools selected as a cluster from February 2010 to July 2010 in Sari, North of Iran. A questionnaire was provided according to International Study of Asthma and Allergies in Childhood (ISAAC) protocol. Asthma, AR, eczema and their combinations were recorded.

**Results:**

Out of the 1818 cases, 646 (35%) subjects had allergic disorder; 223 (12%) had asthma, 318 (17%) had AR and 105 (6%) had eczema The prevalence of allergic disorder in boys (65%) was higher than the girls (40%) (p<0.05).

**Conclusion:**

The results show that around one – third of the elementary school children have allergic disorders. The prevalence in males is higher than the females.

Allergic diseases including asthma, allergic rhinitis (AR) and eczema are common chronic diseases in childhood (1). In the recent decades, the prevalence of allergic diseases such as asthma and allergic rhinitis is increasing in the world, and no specific reason has yet been found for this trend (2). Increasing prevalence of these diseases follows a rapid trend which is most probably due to environmental changes and less commonly due to genetic factors (3). Allergic disorders are a consequence of interaction between genetic and environmental factors. The climate (warm and humid), air pollution, lifestyle, diet and tobacco smoking are risk factors for the increase of allergic diseases (4). Many studies of the population is prevalence of allergic disorders showed international differences (5). The international study of asthma and allergies in childhood (ISAAC) was a standardized methodology to evaluate the prevalence and severity of asthmatic symptoms, rhinitis and eczema among the children and to compare the results among countries (6). 

Therefore, allergic conditions have increased since the past decades, and is posing a heavy burden on health care systems (7). Asthma is a major cause of chronic morbidity and mortality throughout the world. It is well known that rhinitis and asthma coexist in many patients. Allergic rhinitis is a recognized risk factor for asthma, with 20–30% of these patients having asthma, conversely, 60–80% of patients who have asthma have coexisting allergic rhinitis (8). Delayed diagnosis may lead to permanently decreased lung function that could have been prevented by early treatment and it is important that an accurate diagnosis be made as early as possible to access; Minimal (ideally no) chronic symptoms including nocturnal symptoms, no limitations on activities including exercise, and minimal (infrequent) exacerbations. Asthma is a chronic disease that does not have a cure; however, asthma can be controlled (8).

The aim of this study was to determine the prevalence of asthma, AR, eczema and their combinations in the children of elementary schools in Sari, North of Iran. 

## Methods

This cross-sectional study was carried out on elementary school children in the north of Iran (Sari) from February 2010 to July 2010 to determine the prevalence of the common chronic diseases. Totally, 1818 children [1178 boys (65%) and 640 girls (35%)] 7-12 years old were screened in this study. A questionnaire was provided according to ISAAC protocol (5). The diagnosis of the allergic diseases was confirmed by an allergist. We evaluated asthma, AR, eczema and their combinations in our study from 2000 pupils. 1818 questionnaires were collected (91%). The data were collected and analyzed using SPSS 17 and Chi square test. The prevalence of allergic disorders were determined and compared between the males and females. 

## Results

From the 1818 subjects, 1178 (65%) cases were males and 640 (35%) cases were females. The number of cases in the first to the fifth levels are shown in table 1. Out of 1818 pupils; 646 cases had allergic disorder (35%) including; 223 (12%) had asthma; 318 (17%) had AR, and 105 (6%) had eczema (table 2). The prevalence rates of asthma decreased with increasing age but the prevalence of AR increased with age. The total number of allergic boys was higher (65%) than girls (40%) (p<0.05) (table 2). The boys to girls' ratio were 1.5. The AR symptoms are shown in table 3. 

The prevalence rates of eczema in children was 28.8%: males 24.7% and females 36.3%.

**Table 1:** Distribution of Number of elementary school student’s sex in sari, the north of Iran

**Sex** **Level**	**Male**	**Female**	**Total**
First	230	127	357
Second	237	125	362
Third	235	128	363
Fourth	245	127	372
Fifth	231	133	364
Total	1178 65%	640 35%	1818 100%

**Table 2:** Distribution of Asthma, AR, Eczema and combination of them in elementary school student’s according to level and sex in sari, the north of Iran

**Disease** **Level**	**Asthma**		**AR**		**Eczema**		**As+AR**		**As+Ec**		**Ar+Ec**		**As,Ar,Ec**	
	**M**	**F**	**M**	**F**	**M**	**F**	**M**	**F**	**M**	**F**	**M**	**F**	**M**	**F**
First	19	18	27	15	6	3	7	6	3	4	5	5	3	3
Second	19	18	27	21	7	2	7	4	4	4	5	5	2	3
Third	22	10	29	19	10	6	10	7	5	5	8	5	4	3
Fourth	22	10	30	20	6	5	9	8	5	3	7	4	5	3
Fifth	14	7	35	18	6	5	6	4	4	2	4	3	4	2
Total	96	63	154	88	35	21	39	29	21	18	29	22	18	14

As=asthma AR; allergic rhinitis, Ec=eczema

**Table 3:** Prevalence of allergic rhinitis symptoms in elementary school students according to sex in Sari, the North of Iran.

**Sex** **symptoms**	**Male** **N (%)**	**Female** **N (%)**	**Total** **N (%)**
Unilateral nasal symptoms	137 (8)	92 (5)	229 (13)
Bilateral nasal symptoms	196 (11)	107 (6)	303 (17)
PND with thin nasal discharge	197 (11)	83 (4)	280 (15)
PND with thick nasal discharge	212 (12)	131 (7)	343 (19)
Facial pain	61 (4)	49 ( 2)	110 (6)
Loss of Smile	171 (9)	72 (4)	243 (13)
Sneezing	411 (22)	195 (11)	606 (33)
Eye symptoms	96 (5)	71 (4)	167 (9)
Family related allergic rhinitis	232 (13)	172 (10)	407 (23)

## Discussion

 In this study, the 12 month prevalence of wheezing in elementary school children was 13% but the prevalence of asthma was 12%. Allergic rhinitis and eczema prevalence rates were 17% and 5% respectively. The history of total wheezing and the history of wheezing in the last 12 months, were higher in Rasht than in our study, 21.3% and 25.5% in males, 21.4% and 18.4% in females, respectively, but these data in Tehran were 13.6% and 57.5% in males respectively, while in females, were 18% and 58.1% respectively (9). The prevalence of asthma and allergic rhinitis were higher among the boys than the girls in our study which is in accordance with the accredited studies (9-11), but the prevalence of asthma was higher among the female elementary school pupils in Tehran (9) and no specific reason was found in this difference. Therefore, 1/3 of elementary school pupils have allergic disorders. According to Mohammad beigi`s meta analysis, the prevalence of asthma in Iranian elementary schools was 3.9% though our study showed a higher prevalence of asthma that we thought was related to many factors such as high humidity and warm climate. Of course, the socioeconomic, ecological and ethnic factors might have affected the prevalence of asthma in Iran. Similar to our study, the other reports in Iran mentioned that asthma is higher among males than females (10). 

Therefore, further research is required for the comparative evaluation of the social, economical, cultural and most importantly the environmental status of children in Sari to find the scientific and logical solutions of the higher prevalence of asthma among the Sari children compared to other parts in Iran. Not only the prevalence of asthma in our study was higher than other countries such as South Korea, and Thailand which have the same (high humidity and warm) climate but also, it was higher than the south of Iran such as Jahrom, and Isfahan which have dry and warm climate. Also this is similar to other studies in the north of Iran (11-15). 

However, the prevalence of allergic disorders is different between the other countries and our country The AR prevalence was 17% which is lower than Gorgan (in the east-north of Iran) and Bangladesh, but it is similar with Thailand and Babol, because of the similar climate in both regions (12, 15-17). Of course, some believe that allergic disorders are the diseases of the developed countries and several factors are responsible for its increasing prevalence such as inadequate ventilation of closed-environment, nutrition, increased tendency to indoor activities and games, higher exposure to closed-environment allergens, smoking and use of broad spectrum antibiotics (18). The other factors responsible for allergy and asthma are pets, cockroaches, humidity and especially mites and dust (19). We have all of them in our region especially humidity, mites and antibiotics. In our study, eczema was 5% lower than Thailand (7) but higher than Babol (15), and similar to Bangladesh (17) and Croatia (20). 

Like other similar studies, lifetime wheezing (25%) showed the highest prevalence in comparison to all other symptoms regardless of age, because asthma and non asthma disorders caused it. Taking antibiotics (3 times a year) (24%) is high. Sneezing was the most common and facial pain with loss of smelling is the least common symptom in AR patients. In our study in eczema patients, the pruritus is higher than the other symptoms. The distribution of rash was classic, often on the cheeks followed by the elbow and knee extensors. In our study, the prevalence of asthma, AR and eczema is higher in boys than in girls, they are in moderate prevalence as compared to other countries. We suggest further research for the comparative evaluation of social, economical, cultural and most importantly the environmental status of our patients to find the logical solutions of the different prevalence of allergic disorders in different regions.

There are some defects in our study such as; the girls are less than the boys as well as the total number of participants less. Also, we used a wide range of age between 6 to 12 years old. Therefore, we suggest that in the future studies, the total number of participants should be more and also both genders should be at least equal in number. In addition, the range of age must be limited to 6-7 or 13-14 years old. 
